# Gag drug resistance mutations in HIV-1 subtype C patients, failing a protease inhibitor inclusive treatment regimen, with detectable lopinavir levels

**DOI:** 10.7448/IAS.17.4.19784

**Published:** 2014-11-02

**Authors:** Sameshnee Kelly Pillay, Urisha Singh, Avashna Singh, Michelle Gordon, Thumbi Ndungu

**Affiliations:** HIV Pathogenesis Programme, University of Kwa-Zulu Natal, Durban, South Africa

## Abstract

The development of antiretroviral (ARV) drugs and their use in human immunodeficiency virus type 1 (HIV-1) has led to the effective control of HIV replication in infected patients. However the emergence of resistant HIV-1 strains still remains a problem. Literature has shown that mutations may accumulate in the protease (PR) and gag regions of HIV-1 patients who fail therapy with protease inhibitor (PI) drugs [1, 2]. Gag mutations have also been found to play an important role in the evolution of PI resistance [2]. Despite this, the standard genotypic drug-resistance test examines mutations in the reverse transcriptase (RT) and PR region of HIV-1 and not gag [3]. This study investigated the frequency of gag drug resistance mutations in the absence of major PI mutations in HIV-1 subtype C patients, failing a PI inclusive treatment regimen. Sixty-eight samples were retrieved from patients that were classified as second line treatment failures as they had a viral load greater than 1000 copies\mL, as well as detectable lopinavir (LPV) levels. The gag and protease region of these patients were genotyped. Mutations in the gag and protease region were assessed using the REga Db sequencing tool and the CPR programme on the Stanford University HIV drug resistance database. The mean LPV level of these samples was 11.66 µg/mL. 69.11% (n=46) of the patients have no major PI mutations in protease. The following mutations that are associated with PI exposure were present in the data set: G62R (n=6), H219Q (n=11), S737T (n=8), I389T (n=8) and Q474L (n=7). Predictably, mutations that are associated with PI resistance were found, which are generally located in the p7/p1 and p1/p6 cleavage site. These mutations are K436R (n=4), I437V (n=1), L449P (n=5), R452K (n=4) and P453L\T (n=9). These results contribute to the knowledge of resistance mutations in gag and their impact on PI resistance.
